# Quantifying NFT-driven networks in crypto art

**DOI:** 10.1038/s41598-022-05146-6

**Published:** 2022-02-17

**Authors:** Kishore Vasan, Milán Janosov, Albert-László Barabási

**Affiliations:** 1 Network Science Institute, Northeastern University, Boston, USA; 2 Datapolis Inc., Budapest, Hungary; 3 Department of Data and Network Science, Central European University, Budapest, Hungary; 4 Department of Medicine, Brigham and Women’s Hospital, Harvard Medical School, Boston, USA

**Keywords:** Mathematics and computing, Computational science, Computer science

## Abstract

The evolution of the art ecosystem is driven by largely invisible networks, defined by undocumented interactions between artists, institutions, collectors and curators. The emergence of cryptoart, and the NFT-based digital marketplace around it, offers unprecedented opportunities to examine the mechanisms that shape the evolution of networks that define artistic practice. Here we mapped the *Foundation* platform, identifying over 48,000 artworks through the associated NFTs listed by over 15,000 artists, allowing us to characterize the patterns that govern the networks that shape artistic success. We find that NFT adoption by both artists and collectors has undergone major changes, starting with a rapid growth that peaked in March 2021 and the emergence of a new equilibrium in June. Despite significant changes in activity, the average price of the sold art remained largely unchanged, with the price of an artist’s work fluctuating in a range that determines his or her reputation. The artist invitation network offers evidence of rich and poor artist clusters, driven by homophily, indicating that the newly invited artists develop similar engagement and sales patterns as the artist who invited them. We find that successful artists receive disproportional, repeated investment from a small group of collectors, underscoring the importance of artist–collector ties in the digital marketplace. These reproducible patterns allow us to characterize the features, mechanisms, and the networks enabling the success of individual artists, a quantification necessary to better understand the emerging NFT ecosystem.

## Introduction

Despite the important social, cultural, and historical role art plays in the society^1–3^, the evolution of the art ecosystem lacks transparency, being driven by largely invisible interactions between artists, institutions (galleries, museums), collectors and curators. In other words, artistic success is controlled by gate-keepers and informal networks^4,5^, that rely on information that is available only to those with privileged access^6^. In contrast with science, whose main output, the research papers, are publicly available and catalogued in databases, we lack systematic data on the list of works (products) and the transactions that shape the evolution of the art ecosystem. Consequently, while a new discipline called the science of science^7,8^ has emerged to explore the quantitative patterns characterizing science, artistic careers have largely resisted quantification^9^. This has changed recently with the emergence of crypoart, and the cryptocurrency-based digital marketplace born around it, in which all transactions are open and visible. The resulting transparent art ecosystem is accessible to all participants without formal gatekeepers and barriers^10^, offering unprecedented opportunities to quantify and understand the forces, mechanisms, and hidden networks that shape its evolution.

A Non-Fungible Token (NFT) is a permanent and certifiable online record that connects a digital artwork, often called cryptoart, to its owner. Most NFTs are listed on an Ethereum monitored decentralized cryptocurrency platform that utilizes blockchain technology, normally used to power millions of transactions across the globe for multiple applications^11^. Each transaction pertaining to an NFT and the associated artwork is stored in a ledger via a Proof-of-Work (PoW) mechanism, enabling easy and fail-proof transfer of digital assets, and verifiable ownership of art^12,13^. Hence, NFTs^14^ offer a mechanism for artists to create digital works of art and validate their work as unique, eternal, and worth collecting^15,16^, and offers collectors the ability to showcase their collections on digital platforms. Driven by this technological innovation, digital art experienced $2.5 Billion in sales just within the first two quarters of 2021^17^.

The trading of cryptoart is mediated by trading platforms like OpenSea, NiftyGate, SuperRare, Foundation, and others. Previous research on cryptoart has focused on one of the earliest platforms, SuperRare, which is a curated, invitation based cryptoart gallery, exploring the evolution of the marketplace^18^, co-ownership patterns^19^, evaluation by expert art curators^20^, and re-sale dynamics^21^. Here we focus on Foundation (https://foundation.app), launched in February 2021, a rapidly growing artist-driven platform, focused on highlighting the art of recognized artists. In contrast with platforms like SuperRare and NiftyGate, where new artists are accepted slowly through an application process, Foundation is an open platform, meaning that any active artist or collector can invite new artists. This decentralized access has turned Foundation into an organically and rapidly growing marketplace whose evolution mirrors the demand and interest in cryptoart. Given its open architecture, through Foundation we can trace the dynamics of creating, bidding, buying, and selling art, allowing us to map out the complex interconnected network that govern the relationships between artworks, artists, and collectors. Further, it allows us to investigate artistic careers, the emergence and influence of social networks between artists^9,22^, their multiple stakeholders^23,24^, and the patterns that govern the success of individual artists.

### Data collection

Each artwork (NFT) minted on Foundation contains a unique contract address followed by a unique identifier for the artwork (also indexed through tokens). For example, one of its early adopters, *NyanCat*, sold a viral animation of a cat on February 19, 2021, for 300 Ethereum (worth over a million USD today; https://foundation.app/NyanCat/nyan-cat-219). NyanCat’s art can be found on the Ethereum network as contract address 0*x*3*B*3*ee*1931*Dc*30*C*1957379*FAc*9*aba*94*D*1*C*48*a*5405 and token id 219, two pieces of information that allow us to identify all of the artwork minted on Foundation. We used the public open-source Graph API (https://thegraph.com/) to extract the metadata about the artworks, identifying the creator of the artwork, minting and listing time, all the bids (monetary offers to purchase it), allowing us to reconstruct the selling (primary market) and re-selling (secondary market) history of each artwork. This data extraction mapped 50,723 minted artworks, of which 48,059 (94.7%) are listed for sale, and 15,279 (31.7%) have been sold. The listed artworks have collectively received 37,013 bids from 7787 bidders. We find that 1928 (12.61% of all sold) artworks have been re-listed for sale on the platform, and 138 (7.15%) have re-sold. This indicates that Foundation is predominantly used for primary sales, compared to SuperRare, which has a more robust secondary market^19,21^. As a result, we focus only on the primary market, i.e. the listing of the artwork by the artist and its first purchase by a collector.

The artists and collectors on the platform have a unique etherscan wallet id that they use to list and bid for art. We used this id to extract the profile metadata of each user, together with the list of followers (i.e. other users that follow that artist on the Foundation platform), links to social media sites like Twitter and Instagram. We extracted metadata information about 15,366 artists and 5534 collectors on the platform and the twitter metadata for 13,487 (87.7%) artists, including profile address, the number of followers and following. To control for the changing monetary value of Ethereum tokens, we map the ETH bidding amount to its USD price at the day of sale (https://etherscan.io/chart/etherprice). The dataset, extracted on June 18, 2021, is available on a Github repository at https://github.com/Barabasi-Lab/crypto-art, along with the code used to crawl the data.

## Results

### The rise and the fall of the NFTs

**Figure 1:**
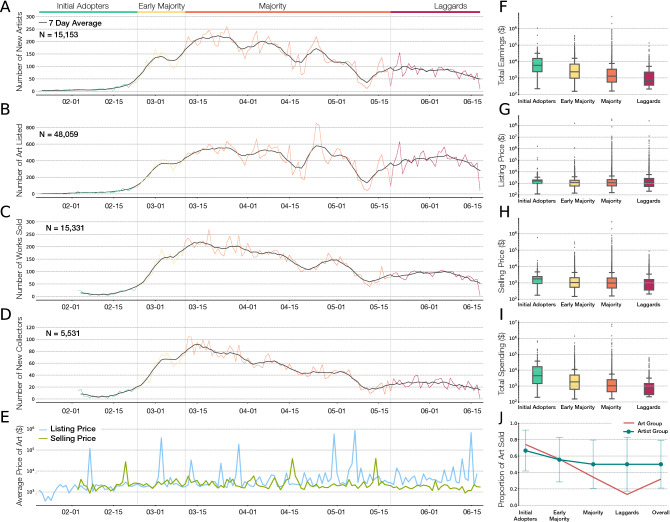
The timeline of NFT adoption on foundation. (**A**) Number of new artists listing art, indicating that many artists joined the platform in March and that adoption reached a new equilibrium following a decline in May. (**B**) Number of new art listed on the platform. (**C**) Number of artworks sold on the platform. (**D**) Number of new collectors purchasing art. (**E**) The daily listing and selling price of art, indicating that despite the changes in NFT adoption rate, the economic value of art has remained unchanged. (**F**) The earnings of innovators, early majority, majority, and laggards, showing that early artists have enjoyed higher earning than late joiners. (**G**) The list price of art in the four artist groups. (**H**) The selling price of art grouped by selling date. (**I**) The spending of collectors within each group, indicating that early collectors have been higher spenders than the late comers. (**J**) The selling rate of art within each artist and art group, showing that artworks listed in early groups have sold at a higher rate than in the late stages. Error bars show the standard deviation.

To understand the temporal and historical dynamics of cryptoart, we begin by investigating the patterns of activity of the Foundation platform, focusing on new artists (Fig. 1A), art listed (Fig. 1B) and sold (Fig. 1C), and the arrival of new collectors (Fig. 1D). The data indicates that the adoption of digital art exploded in late February 2021, peaked during March, and found a new steady state around May 2021. This pattern is particularly obvious if we inspect the arrival of new artists, indicating that the platform attracted an exceptional number of new artists in mid March and April, following Christie’s attention-grabbing auction of Beeple’s art for $69,346,250^25^. However, early May the number of new artists briefly dropped back to its early-March level (Fig. 1A), raising again to reach a new steady state. This rise and fall is also well documented by the number of art sold and number of first-time collectors on the platform (Fig. 1C, D), curves whose temporal trends strongly correlate with the number of new artists.

This adoption timeline allows us to investigate the patterns of success within each group, defined by the time when each artist/collector listed/bought art on the Foundation platform. While new artists and collectors continue to join the platform, they do so in smaller numbers, and the desire to purchase large number of artworks within short periods of time has faded, allowing the system to reach a new steady state activity level. Note, however that the decline in the adoption of new artists and new collectors in May does not imply a return to the pre-March attention to NFT’s as there are 13 times more artists and 12 times more collectors on the platform on June 1 than there were on March 1. Rather, the saturation of the adoption process suggests that most artists and collectors that intended to adopt in the first wave of the NFT art movement have already joined the platform.

### First movers’ advantage in sales and valuation

Innovation research classifies the adoption of new technologies into five temporal stages^26,27^: (1) Adoption by innovators (or initial adopters), which in our case corresponds to the first 2.5% artists/collectors who joined the platform (from 21 January, 2021 to 22 February, 2021); (2) Arrival of early adopters (or early majority), representing the next 13.5% (from 23 February to 10 March); (3) Emergence of majority (34%) and late majority (34%) between 11 March to 18 May (4) Adoption by laggards, representing the final 16% (19 May to 18 June). The earnings of artists and the selling price of art in the different adoption stages helps us understand the advantage of the early adopters. Indeed, we find a monotonic decrease in total earnings with adoption time, indicating that artists who joined the platform later have earned considerably less than the artists that entered early (Fig. 1F).

Similarly, collectors who joined the platform later have spent less than the early adopters (Fig. 1I). These trends, however, may only represent a cumulative effect: those who arrived at an earlier time had more opportunities to sell or purchase art. Yet, if we normalize by time on platform, the first movers’ advantage persists: initial adopters have earned more than the early majority, which in turn earned more than the laggards, a pattern characterising collector spending as well (SI Fig. S2B, F). Early artists have also sold more art for a higher average price than the late comers’ (SI Fig. S2A, C, Normalized: D), and early collectors have invested in more art at a higher price than new collectors (SI Fig. S2E). This indicates that early adopters have established themselves as successful digital artists and collectors, benefiting from a first-mover advantage^28,29^.

Interestingly, the rise and the decline of interest in NFTs (Fig. 1A–D) has not affected the average listing or selling price of the artworks: the daily listing and selling price has stayed remarkably stable over the explored five month period (Fig. 1E; for grouped counts see Fig. 1G, H). In other words, the value of the art has remained largely unaffected by the major influx of artists and collectors in the NFT space, and the complex temporal dynamics of the platform. Furthermore, the time it takes to sell an artwork also remained unchanged in this period (SI Fig. S4). What has changed is the likelihood that a listed artwork does sell, measured as the fraction of the sold inventory (Fig. 1J): 74.1% of the art released during the innovator period has found a collector, but only 13.3% of the art released during the laggard period sold.

Overall, we find that new artists have a diminished ability to sell art due to two connected factors (1) there is a considerably large inventory on the market, making it difficult for new artists to attract the attention of collectors and (2) the urge to buy digital art has stabilized. It is rather remarkable, however, that this complex market dynamics has not affected the listing and the selling price of art.

### In-platform followers shape valuation

On Foundation, collectors can keep track of their favorite artists and their new art by following them, hence the number of followers of an artist captures the collective interest in an artist’s work. Most artists are also present on Twitter, and many of them regularly tweet their new work released for sale on Foundation. This raises several important questions: to what degree does visibility on social media, like Twitter, translate into visibility on the Foundation platform, as measured by the number of Foundation followers? Does external (Twitter followers) and internal (Foundation followers) visibility affects the value of the art and the likelihood to find a buyer?

**Figure 2:**
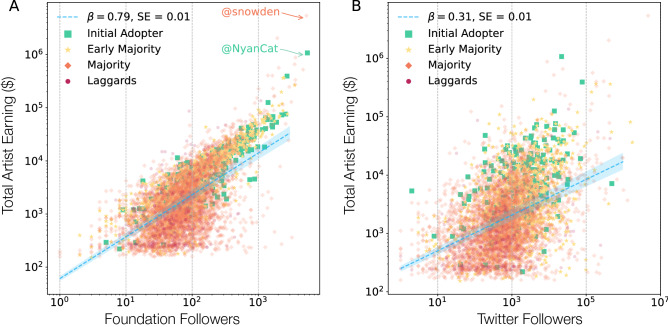
The impact of follower count on artist earnings. We measure the number of followers on Foundation (internal) and Twitter (external) for each artist to measure the role of visibility on artist earnings. (**A**) The number of Foundation followers vs the total artist earning, finding a sublinear scaling with exponent $\beta = 0.79$ (CI 0.76–0.81; $R^2 = 0.41$), indicating that the more foundation followers an artist has, the higher the auction price of his/her work. Unsurprisingly, the top earning artist on Foundation, *NyanCat*, also has the most followers on the platform. (**B**) The number of Twitter followers and the total artist earning, finding that Twitter is a weaker indicator of artist earning, with a sublinear scaling of exponent $\beta = 0.31$ (CI 0.28–0.32; $R^2=0.18$). These results indicate that artist success depends on both platform and outside visibility, but measures of external following have weaker impact compared to the internal on-platform following.

To answer these questions, we measured the number of followers an artist has on the Foundation platform (internal) and the number of followers on Twitter (external). To test the effect of this visibility on sales, we quantified the impact of the number of followers on an artist’s earnings (see SI 4). We find that the total artist earning grows as $$N_{followers}^{\beta }$$ with $$\beta = 0.792$$ (CI [0.768–0.816]), capturing a strong sublinear growth with the number of Foundation followers (Fig. 2A). We find a particularly strong correlation with the maximum price an artist receives for his or her work (Foundation $$\beta = 0.935$$). For example, *NyanCat* with 5530 followers on Foundation has sold 5 artworks for a total of $1,078,247. At the same time the Twitter follower count is a weaker indicator of artist earning ($$\beta = 0.305$$; CI [0.288–0.322]), indicating that visibility on Twitter doesn’t translate into earnings on Foundation (Fig. 2B). In other words, prestige and the earning power of an artist is derived primarily from his or her visibility on the Foundation platform.

### The social network of the artists

**Figure 3:**
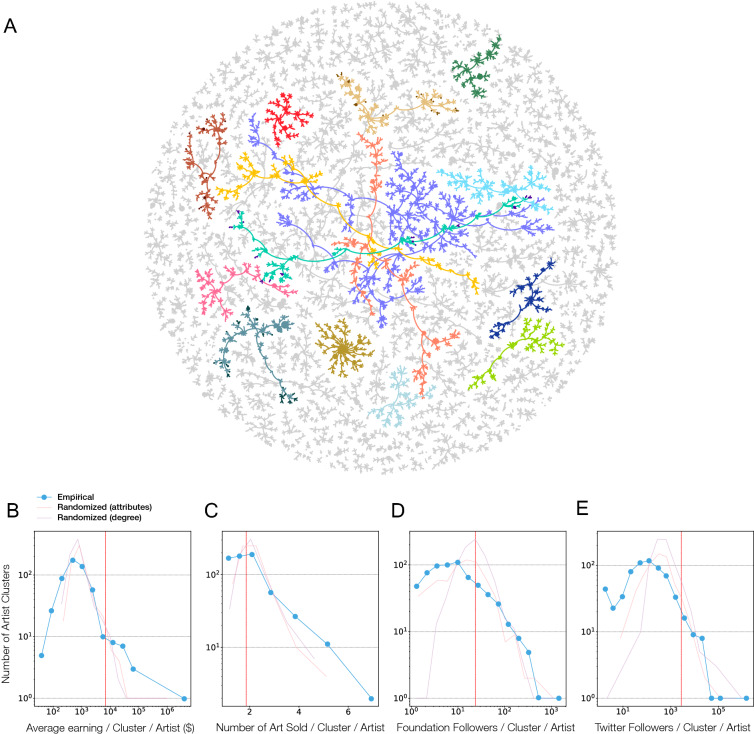
The artist social network. (**A**) The first 100 days of artist adoption through invites. Each node corresponds to an artist and a directed link connects an artist to the artist who invited him or her to the platform. We show all clusters with more than 10 artists, resulting in a total of 13,010 nodes and 204 clusters. The top 20 artist clusters in terms of number of artists are colored and nodes are sized based on the number of artworks sold. The largest component (purple) consists of 941 nodes. To characterise the artist clusters, we perform two types of randomization, the first involves shuffling the node attributes and the second involves degree preserving link randomization. (**B**) The distribution of average earning per art per artist cluster, finding the emergence of poor and rich clusters, both extremes absent in the random reference data. (**C**) The distribution of number of art sold per artist per cluster, finding that artists in some clusters sell a lot more art than expected by chance. (**D**) Number of Foundation followers per artist per cluster and (**E**) Number of Twitter followers per artist per cluster, finding the existence of low and high popularity within communities, extremes absent in the random reference set. Red line indicates the average value of each of the empirical distributions.

Foundation is an organically growing platform, as it allows artists who have listed art on the platform and collectors who have purchased art to invite new artists. Information of who invited whom is displayed on each artist’s page, allowing us to map out the social network that fuels the adoption of the platform by new artists. Defining a node as an artist and a directed link marking who invited the artist to the platform, we obtain an artist network with 14,706 nodes (artists) and 14,066 links (invitations), which is fragmented into 640 isolated components (artist clusters) with an average community size of 22. Each artist cluster forms a tree, and we find that 594, or 91% of the clusters have emerged within the first 100 days of the platform, each containing on average 16 artists (SI Fig. S6). These early artist groups initiated the subsequent growth of the platform (SI Tab S2), inviting further artists (Fig. 3A).

For example, the largest artist cluster has 941 artists (9.26% of all), initiated by the artist *sergeposters*, who listed his first artwork on February 6, 2021, and invited 10 other artists, which in turn invited 43 others, who then invited 86 others, and so on, resulting in a cascade of adoptions. The resulting artist cluster collectively is responsible for $1,661,173 in sales from 867 artworks, raising the question if the artists’ success in selling art depends on the cluster she joined. We therefore measured the distribution of the average earning per art per artist in each cluster, finding it to be skewed to the right, indicating the presence of a few clusters whose earnings is much higher than expected if the artist clusters formed at random (Fig. 3B). Indeed, in the empirical network, the maximum earning cluster collected $2,703,171 (with a mean of $6766), while in the randomized reference, where we shuffle the node attributes and conduct a degree preserving link randomization, the largest earning cluster has $901,796 (with a mean of $2684). Such high-earning clusters continue to persist if we disregard the highest earning artwork within each cluster (SI Fig. S8). In the opposite limit, we also find evidence of “poor clusters”, i.e. clusters whose artists earn less than expected by chance. Taken together, we observe a segregation into both rich and poor artist communities, extremes absent in the random reference data. The differences between the artist clusters are also captured by multiple measures, from the number of artworks sold (Fig. 3C) to the number of Foundation followers (Fig. 3D), and the number of Twitter followers (Fig. 3E).

Finally, we find that the differences between artists and their invitees in terms of earnings, number of art sold, and followers to be minimal, evidence of a strong homophily at the individual artist-invitee level (SI Fig. S7). In other words, the earnings of a new artist tend to be similar to that of its invitor, offering evidence that the social network of artists is built through a unique combination of perceived reputation and follower base, that leads to a self-organized emergence of clusters that subsequently determine the success of individual artists.

### Patterns of artist success

**Figure 4:**
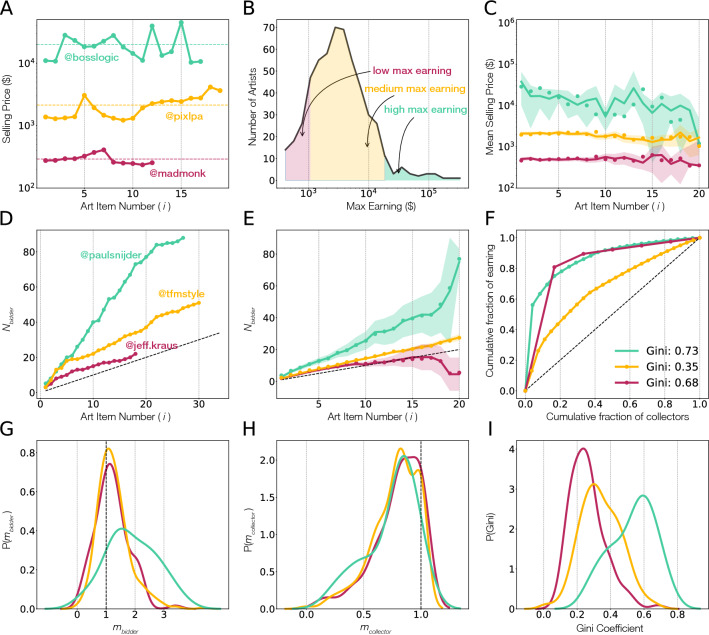
The emergence of artist reputation. (**A**) The price of subsequent art sales (*i*) for three artists, *bosslogic*, *pixlpa*, and *madmonk*. The lines indicate the average sales price for each artist. (**B**) We grouped artists who sold more than 5 artworks based on their max selling price, allowing us to distinguish high reputation (green, top 5%), medium reputation (yellow, middle 75%) and low reputation (red, bottom 20%) artists. (**C**) The evolution of the selling price of an NFT as a function of the art item number for high, middle and low reputation artists. Symbols indicate the true observations while the shaded area indicates the random reference, obtained by randomizing artists careers (95% confidence interval). (**D**) The growth in the cumulative number of collectors ($N_{bidder}$) bidding for the work of three artists, *paulsnijder*, *tfmstyle*, and *jeff.kraus*. (**E**) The growth in the number of bidders ($N_{bidder}$) for different artist groups, highlighting the differences in growth rates based on artist reputation. Symbols indicate the true observations and the shaded regions represent the randomized careers (85% confidence interval). (**F**) The inequality curve (Gini coefficient) of investment by different collectors for the work of the same three artists as in (**D**). We quantify the linear growth in the collector base by measuring the slope of the rise in cumulative number of bidders ($m_{bidder}$) and collectors ($m_{collector}$). (**G**) The density function of $P(m_{bidder})$ signalling bidder growth rate. (**H**) The density function of $P(m_{collector})$ showing collector growth rate. (**I**) The Gini coefficient distribution of artists for different artist groups. The coefficient ranges from 0 (perfect equality) to 1 (perfect inequality), demonstrating that the investment by collectors for high reputation artists is highly unequal.

In the classical art market, the price of the artworks and the number of sold works grows with the artist’s increasing reputation. Some of this growth is maintained by galleries, who hold the price of art even when demand drops. As the NFT space lacks galleries as gatekeepers, we were curious if reputation effects have naturally emerged. We ask therefore, what is the impact of the previously sold art on the future price of the work by the same artist. Interestingly, the price history of the top selling artists fails to indicate an increasing trend in the art price (Fig. 4A). Rather, it is not uncommon to have significant differences between subsequent sales (SI Fig. 12). For example, a high selling artist *Allo* sold his/ her fifth artwork for $2028, while the subsequent works fetched only $274, $332, $647, $818 respectively, while the tenth art selling again for $2178. Similarly, *bosslogic* sold his/ her eighth artwork for $27,987, but the subsequent works found lower sales of $18,165, $14,443, $11,082, until the twelfth work sold at much higher price point of $39,708 (Fig. 4A, green).

Despite these fluctuations, we also observe a certain degree of stability, finding that for most artists the sales price fluctuate in a well-defined range. For example, *bosslogic* repeatedly attracts sales in the range of $10,000 to $30,000 (Fig. 4A, green), while *pixlpa* sells art in the range of $1000–$2000 (Fig. 4A, blue). In contrast, for *madmonk* the sales prices are regularly much lower, in the range of $250–$350 (Fig. 4A, orange). In other words, while sales prices do show considerable variability, they fluctuate within a predictable range that defines the reputation of an artist (SI Fig. 9).

To quantify the role of reputation in the NFT space, we categorized all artists into three groups based on their highest individual sale price: the bottom 20% (low reputation, top sale being less than $1254), the middle 75% (medium reputation, top sales between $1254 and $18,510), and the top 5% (high reputation, highest sale above $18,510) (Fig. 4B). The three artist groups are also separated based on number of followers indicating that reputation correlates with popularity (SI Fig. 10). We find that artists within each of these groups experience only nominal changes in their sales patterns, i.e. high performing artists continue to receive higher number of bids for new art (SI Fig. 11) and repeatedly attract high prices for their new art, while low reputation artists have difficulty demanding higher prices (Fig. 4C). In summary, the sales price of the art by the same artist shows a degree of stability over time, indicating the emergence of reputation effect in crypto art, similar to those observed in scientific careers^30^.

### The impact of sustained artist–collector ties

In the traditional art space, collectors discover new artists through gallery and museum exhibits. In turn, gallerists work with curators to create opportunities for emerging artists to showcase their work, both in the galleries and in museums^9^, bringing it to the attention of collectors. As the cryptoart space lacks the formal role of gallerists, curators and museums, success in this space is mediated by potential direct relationships between artists and collectors. We therefore ask, what is the role of collectors in the success of individual artists? We find that the continued activity of an artist attracts collector attention: the number of bidders interested in a given artist’s work grows with the number of sales by the artist (Fig. 4D, E). We approximate the linear growth rate of an artist’s collector base measuring the slope of the cumulative count of bidders ($N_{bidder} \propto m_{bidder}*{i}$) and collectors ($N_{collector} \propto m_{collector}*{i}$), where *i* is the art item number, finding that 80% of the artists have $m_{bidder}>1$. The distribution of $P(m_{bidder})$ documents the explicit differences between artist groups: for high reputation artists the peak is at $m_{bidder} \sim 2$, while for low and middle reputation artists it peaks at $m_{bidder} \sim 1$ (Fig. 4G). In other words, high reputation artists, attract bidders twice as fast than low and medium reputation artists.

At the same time, 75% of the artists have $m_{collector}<1$ (Fig. 4H), a slower growth rate indicating that each subsequent sale does not necessarily attract a new collector, evidence of return purchases by repeat collectors (SI Fig. 13). In contrast with the difference in bidder growth among artist groups, the distribution of collector growth, $P(m_{collector})$ are indistinguishable for low, middle, and high reputation artists, indicating that new and returning collectors follow a common pattern. This similarity prompts us to ask, are there differences in investment patterns of collectors based on artist reputation?

Interestingly, we find that level of collector’s investment across artists is highly uneven (Fig. 4F, I). We quantify such disparity using the Gini coefficient, that ranges from 0 (complete equality, when all collectors of an artists have spent the same amount on the artist’s work) to 1 (extreme inequality, when a single collector buys all of an artist’s work). We find that high reputation artists have an average Gini coefficient of 0.53, much higher than the value obtained for medium (0.34) and for low reputation (0.26) artists, indicating that high reputation artists derive their earnings primarily from a few collectors who make large and repeat investments, while medium and low reputation artists capture a more uniform spend from their collector base (Fig. 4I).

Such artist–collector dynamics is well illustrated by the sales of *PaulSnijder* ($$m_{bidder} = 3.19, m_{collector} = 0.82$$), who has three (12.5% of all) returning collectors that purchased six of his artworks (22.2% of all), spending on them $46,842, representing 61.5% of the artist’s total earnings. The remaining 21 (87.5% of all) collectors account for only 38% of the artist’s earnings. In general, for the top 180 artists who sold 10 or more artworks, the combined 2743 artworks sold by them have collected $5,911,037 from 1264 collectors. Yet, we find that 414 (32.75%) of these collectors made repeated investment, being responsible for 1893 (69.01% of total) artworks for a total of $4,495,120 (76.04% of earnings). Similarly, one of the biggest art collector on the platform, *3FMusic*, invested $5,835,379 in 316 art works from 210 artists, focusing much of their portfolio on repeated investment worth $3,106,445 (53.2%) for 155 (49.05%) artworks from 49 (23%) artists. This indicates that collectors tend to specialize on a small group of preferred artists, spending more than half of their total budget on them, at the cost of limiting the diversity of their collection.

**Figure 5:**
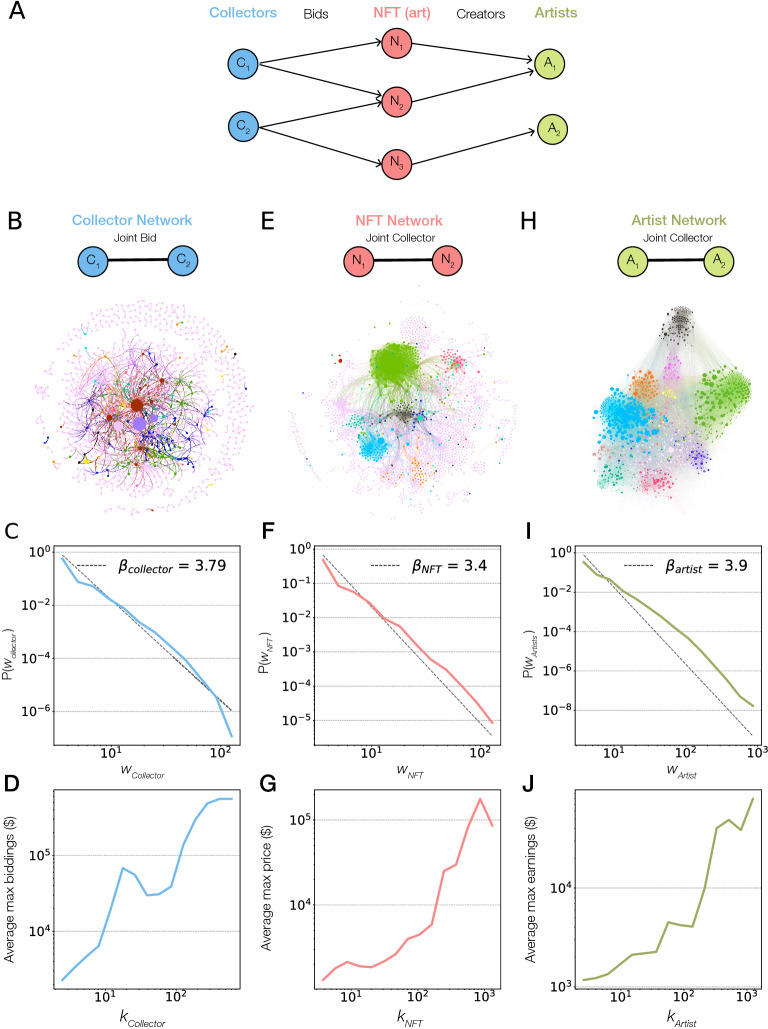
Co-bidding networks. (**A**) Schematic description of various co-bidding networks based on NFT transactions extracted from the data, capturing how a collector $C_1$ bids on an NFT $N_1$ created by artist $A_1$. As collectors can bid on multiple artworks, we use these joint bids to reconstruct the collector–collector, NFT–NFT and artist–artist networks. (**B**) The collector network has collectors as nodes and their joint NFT bids as links. The nodes are sized based on maximum investment and the top 10 collectors are highlighted while other collectors are colored in pink. (**C**) The distribution of edge weights, representing competition, follows a power law decay $P(w_{collector}) \propto w_{collector}^{-\beta _{collector}}$ with exponent $\beta _{collector} = 3.7$, measured using *plfit*^31^. (**D**) The association of maximum bidding amount and degree, finding that highly connected collectors make higher bids. (**E**) The NFT network, where a node is an NFT, connected if the same collector has bid on both the NFTs. The node sizes correspond to the selling price and the NFTs bid by the top 10 collectors are highlighted. (**F**) The distribution of link weights, representing collector similarity, follows a fat tail decay with exponent $\beta _{NFT} = 3.4$. (**G**) The association of connectivity and selling price, finding that central NFTs attract higher prices. (**H**) The artist network, whose nodes are artists and links correspond to joint collectors. The nodes sizes reflect the total earnings of the artist. We color the artists based on bids by top 10 collectors while the rest are colored pink, highlighting the stratification of artists among collectors. (**I**) The distribution of edge weights, representing similar collector interest, follows a fat tail decay with exponent $\beta _{artist} = 3.9$.

These results highlight that artist–collector ties play a crucial role in the earnings of an artist, suggesting that the success for NFT artists rests on their ability to build relationship with collectors that are willing to repeatedly purchase art from them, rather than attracting new collectors. These strong artist–collector bonds are similar to the collection patterns observed in the classical art world, where collectors often focus on a few artists. The evidence of such close ties between artists, collectors, and artworks prompts us to systematically map out the co-bidding networks that define success in crypto art (Fig. 5A), resulting in three maps capturing the collector network, the art network, and the artist network.

### Co-bidding collector network

Collectors are connected to each other if they show common taste and collector philosophy, revealed by frequent joint bids on the same art. Such bidding wars define a co-bidding collector network, whose nodes are collectors and the weighted links between them corresponds to the number of times two collectors bid on the same artwork (Fig. 5B). The resulting network has 7787 collectors as nodes and 20,417 weighted links with an average degree of 5.2. The collectors are fragmented into 2235 components, the largest component accumulating 5223 collectors (67.07%). The number of joint bids (edge weight) determines the competition (or rivalry) between the two collectors. We find that 54.98% of the links between collectors have weight larger than one, evidence of repeated common interests. Furthermore, we find that the link weights are well approximated by a heavy tailed distribution, $P(w_{collector}) \propto w_{collector}^{-\beta _{collector}}$, with exponent $\beta _{collector} = 3.7$, indicating the presence of remarkably strong links between some collectors, corresponding to over a hundred competing bids (Fig. 5C). We observe the highest rivalry between collectors *HypnoPizza* and *3FMusic*, who have bid against each other on 145 artworks. Finally, we find that having a higher degree in the collector network implies higher investment in art (Fig. 5D), indicating that the buying power is concentrated on the few hubs of the collector network who tend to make considerable investments in cryptoart.

### Co-bidding NFT network

The financial success of an NFT is partly determined by its visible features like style, design, content, attracting the interest of collectors with similar taste in art. To uncover potential NFT-NFT connections, we mapped out the co-bidding art network, whose nodes are artwork and a weighted link corresponds to the number of shared bidders (Fig. 5E). The resulting network is particularly dense with 15,338 NFTs connected via 547,566 links corresponding to shared bids. We find that 82.4% of the NFTs are part of a giant component. The link weights are well approximated by a heavy tailed distribution, $P(w_{NFT}) \propto w_{NFT}^{-\beta _{NFT}}$, with exponent $\beta _{NFT} = 3.4$ (Fig. 5F), again evidence of very strong links between few artworks. We find once again that the centrality of an NFT in this network, as captured by its degree, is strongly associated with its selling price (Fig. 5G).

### Co-bidding artist network

Two artists are connected if collectors show simultaneous interest in the work of both artists. Such repeated links may indicate formal, conceptual or stylistic similarities between the two artist’s work. We therefore reconstructed the co-bidding artist network, whose nodes are artists and the weighted links represent the number of shared collectors (Fig. 5H). The resulting network of 6126 artist ties has an average degree of 91.97. The network has a giant component, consisting of 82.35% of the nodes. The edge weight, capturing the strength of common interest by collectors, follows a fat tailed decay, $P(w_{artist}) \propto w_{artist}^{-\beta _{artist}}$, well approximated by a power law with decay exponent $\beta _{artist} = 3.9$ (Fig. 5I). The artists *zawada* and *PaulSnijder* appear to have the highest weight link between them, having received bids from the same 833 collectors for 36 artworks. We find that the degree of an artist in the artist network shows a strong correlation with the artist’s earnings: hub artists earn more for their art (Fig. 5J) than the more peripheral artists.

## Summary and discussion

While the traditional art space is at best opaque, as prices and even the act of a sale is often shrouded in secrecy, the transparent nature of cryptoart offers a historical opportunity to quantify and understand the processes that determine success in the art space. Taking advantage of this open data, here we mapped out the history of a prominent NFT marketplace, the *Foundation* platform. The data allowed us to explore the auction dynamics, artist–collector ties, and co-bidding networks within this self-contained crypto art ecosystem. We find strong evidence of first movers’ advantage, in that innovators and early majority artists had higher earning than the latecomers, and collectors who joined early have also been more engaged, spending more on art.

Examining the career of artists, we find that the price of artworks remains comparable throughout an artist’s career and its range defines the artist’s reputation and popularity. Yet, there are significant fluctuations in art prices for the same artist, a striking contrast to the patterns seen in the classical art world where the price of seasoned artists increases as they gain visibility, and price drops are avoided by galleries. We classify the reputation of artists into low, medium, and high categories based on the maximum price of the sold artwork, finding that high quality artists repeatedly attract high prices for their works, evidence of persistent market-driven reputation effects.

Just like in the classical art space, we find that artist–collector bonds play an important role. Indeed, collectors tend to develop a unique taste in digital art, prompting them to make repeated purchases from a small group of artists. Consequently, the earnings of high reputation artists is ensured by multiple, disproportionate investments by a small group of collectors. In contrast, low reputation artists struggle to attract a sustained collector group, leading to low, uniform investments from several collectors. In other words, the careers of successful artists is built by attracting collectors willing to make repeated investment in their art, rather than appealing to the larger set of collectors investing in crypto art. We show that links between collectors, art, and artists affect the price of art, finding that the value of NFTs is driven by strong network effects between artists and collectors.

Our work also identified important avenues for future research. For example, many important quantities, like the number of on- and off-platform followers, are not encoded in the block chain, but are stored on the Foundation platform, hence we are unable to reconstruct their temporal evolution. For example, we do not know when a collector starts following an artist. The lack of such temporal information limits our ability to unveil causal effects, like the potential causal impact of rich artist groups on the performance of new artists, or how the number of in-platform followers drives art valuation. For such studies to be possible, either the platforms need to supply temporal information for research purposes, or the research community must launch a temporal observatory that monitors all the platform variables in real time.

It wasn’t until a few months ago that the use of NFTs to share, collect, and trade art became a mainstream phenomenon. The coming years, relying on data transparency of the sector, will help explain the influence of NFTs in shaping culture and art, in the same way that physical art are deeply embedded in narrating historical events. As the system establishes itself as a powerful medium of artistic expression, teasing out the networks that drive art prices and artist success will be of the utmost importance.

In many ways, this and other similar works^10,12,13,21^ are only the beginning of a larger research program needed to explain the multi-faceted impact of NFTs on art and society. Many pertinent questions remain to be addressed, like the difference between single versus multiple editions of the same artwork, the role of generative art, and most importantly, how NFT artists build a community of collectors, and how do these community effects shape the careers of an artist. The underlying reproducible patterns identified here, pertaining to artists, collectors, and bidding patterns, demonstrate how data and network science tools can help unveil the processes governing the emergence of the NFT ecosystem.

## Supplementary Information

Supplementary Information.
