# Supplementary material for: Quantifying NFT-driven networks in crypto art
Source: Sci Rep. 2022 Feb 17;12:2769. doi: 10.1038/s41598-022-05146-6 (PMC8854720; doi:10.1038/s41598-022-05146-6)
Supplement: Supplementary file 1 — Supplementary Information. [file 41598_2022_5146_MOESM1_ESM.pdf]

Quantifying NFT-driven networks in crypto art

Kishore Vasan[1], Milán Janosov[2, 3], and Albert-László Barabási[1, 3, 4 *]

[1]Network Science Institute, Northeastern University, Boston, United States
[2]Datapolis Inc., Budapest, Hungary
[3]Department of Data and Network Science, Central Eastern European University, Hungary
[4]Department of Medicine, Brigham and Women's Hospital, Harvard Medical School, Boston, United States
[*]e-mail: a.barabasi@northeastern.edu

Supplementary Information

1 Data

The data for this work comes from *Foundation*. We used the Graph API (https://thegraph.com) to extract 50,723 artworks on the platform. Each artwork has a creator and a collector. Of all the art minted on the platform, 48,059 are listed for sale, and 7,787 works have been sold to 5,534 collectors. The dataset was extracted on June 18, 2021, and we provide the code to conduct reproducible extractions at our github page, https://github.com/Barabasi-Lab/crypto-art. We present the descriptive counts of the data in SI Fig 1.

2 First movers' advantage

We examine the first movers' advantage from the perspective of time on platform. We show the number of art sold and bought by each of the grouped category, along with the average price of art (SI Fig 2). While the earning per art is similar across the artist category, the artists in the innovators and early majority period have sold more art, normalized by time on platform, than the late-joiners. In a similar manner, collectors in the innovators and early majority groups have collected more art, normalized by time on platform. In other words, artists and collectors who adopted the platform in the early stages have sold more and bought more art at a higher rate those who joined later.

3 Bursty effect of auctions

A unique feature of the openness of the cryptoart space is the ability to follow the bidding dynamics and trace the trading history of NFTs. The auction rules of the platform stipulate that each art work must

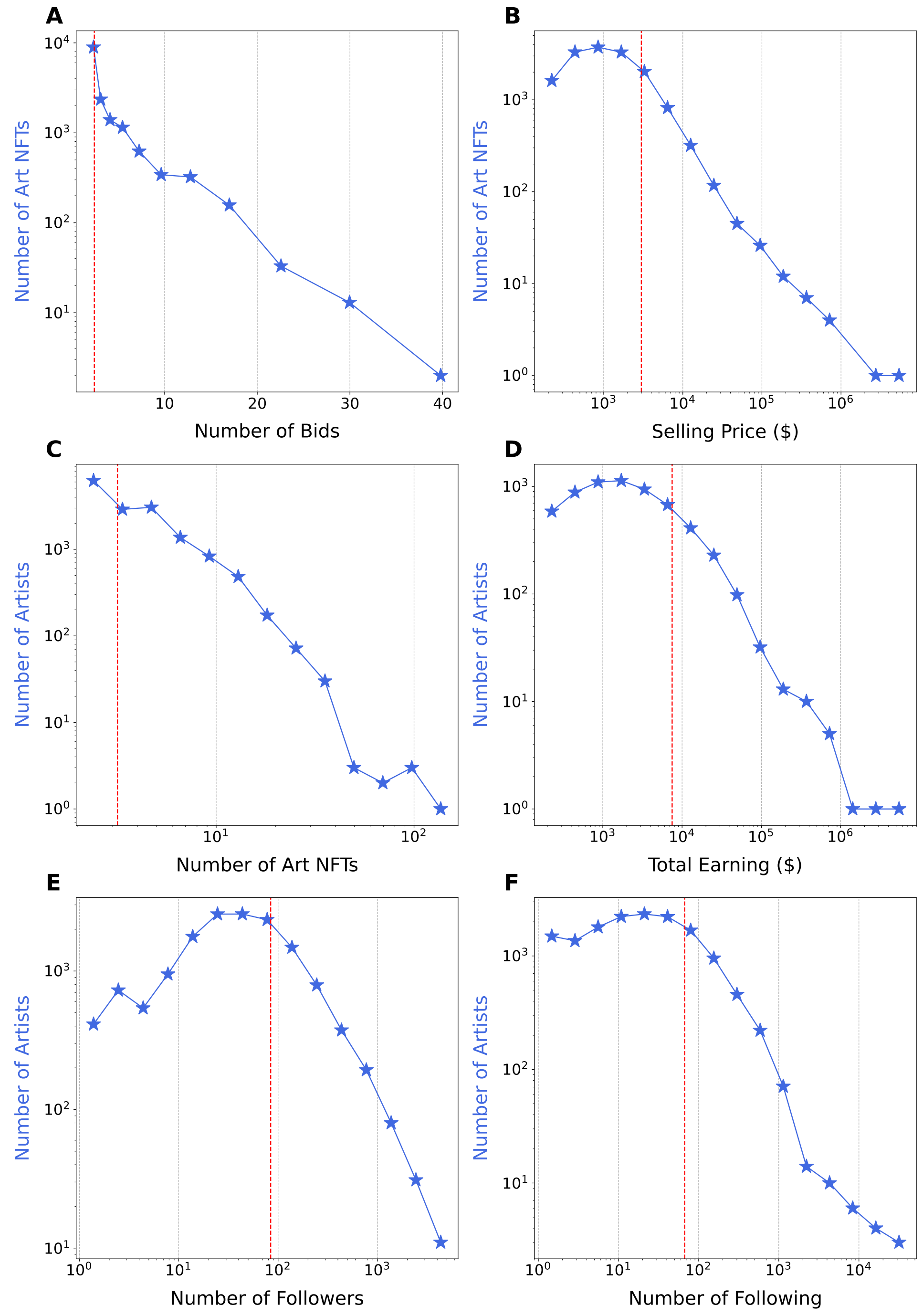

Figure 1. Descriptive counts of the data. (A) The distribution of number of bids for NFTs (B) The distribution of selling price at the time of sale. (C) The distribution of art NFTs listed by artists. (D) The distribution of total earning (E) The distribution of number of followers (F) The distribution of number of following. Red line indicates the average value of each distribution.

be on sale for a minimum of 24 hours after the first bid has been placed and a bid placed during the final 15 minutes of this 24-hour period increases the auction time by an additional 15 minutes. Here, we investigate the changes in price and attention dynamics to the art within this 24+ hour window.

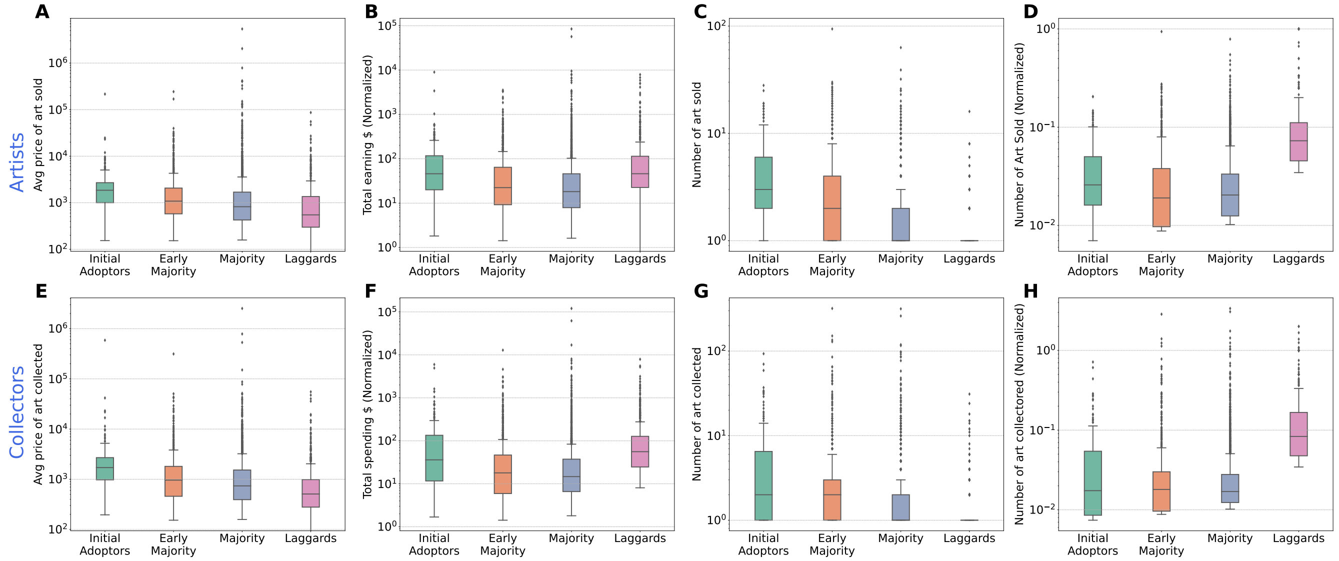

Figure 2. Normalized effect of first movers' advantage. (A) Average price of art sold by artists. (B) Total earning of artists normalized by their time on platform. (C) Number of art sold (D) Number of art sold normalized by their time on platform. (E) average price of art bought by collectors. (F) The total spending of collectors normalized by their time on platform. (G) Number of artworks collected by collectors (H) Number of artworks collectors normalized by time on platform. We find that initial adopters and early majority groups have enjoyed the first movers' advantage in cryptoart.

An examination of the most contested art sold on Foundation (measured by the total number of bids), indicates that most of the bidding took place in the final hour of the bidding timeline (SI Fig 3 A-C). For example, the NFT titled *nfts-explained-12012* (https://foundation.app/visualizevalue/nfts-explained-12012) collected 21 bids from 16 collectors in the final hour of the auction, hence pushing the price of the art from a mere \$4,171 to \$123,483 (SI Fig 3 A). Although a few collectors made bids soon after the beginning of the auction, during the first 18-22 hours only one new bid was placed, and a bidding war emerged only during the final hour of the auction.

Such patterns of bursty biddings are prevalent across all sold art: the bids come either at the beginning of the auction (a requirement to begin the auction) and at the end of the auction (SI Fig 3 D). As a result, we observe a gradual (and necessary) increase in the auction time for the art that achieves the highest bids (SI Fig 3 E) and as expected art sold at the rear end of the auction timeline fetches higher prices (SI Fig 3 F).

Further, new digital art appears to follow an exponential decay in interest from collectors (with decay exponent $\lambda = 0.168$). We find that 33.3% of the art receive the first bid within 12 hours of listing and 52.83% receives its first bid within the first two days (Fig 4 A, B). These bidding dynamics also affects the price of the art (Fig 4 C, D). On average, an art that receives a bid within the first two days sells for

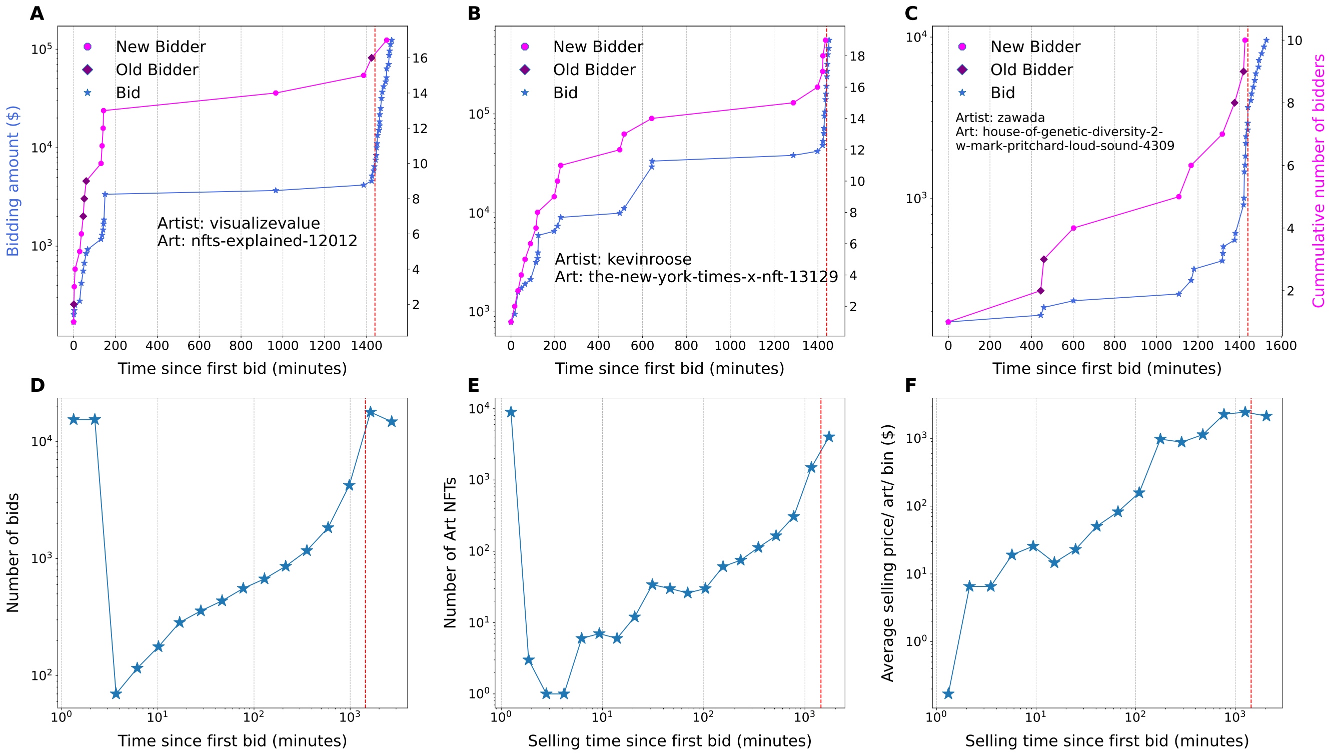

Figure 3. Auction dynamics of art. (A-C) The number of bids, bidders, and bidding amounts over the 24 hour auction window for three different artworks listed by *visualizevalve*, *kevinroose*, and *zawada*. We find that these items saw most of the transactions during the final hours. (D) Number of bids received during the auction duration, indicating that majority of the bids take place during the first hour and the last hour of the auction. (E) Number of NFTs sold as a function of time since first bid, indicating that majority of the artworks is sold either right after the auction begins (i.e. receives very little attention) or during the rear end of the auction window. (F) Average selling price of art as a function of selling time, indicating that art items that have had their auction last for a long time also fetch higher prices.

$4,276, while an art that receives a bid during the third and fourth day receives $1,628. Taken together, these findings demonstrate the bursty activity of cryptoart, indicating that artworks sold at higher prices (1) feature bidding wars towards the rear end of the auction and (2) the bidding tends to start during the first two days of listing, taking advantage of the novelty of the listed art.

4 Estimates for scaling coefficient

We estimate the association of followers and their total earning by estimating the scaling coefficient on the log scale regression. First we estimated the overall scaling across all artists and then for each group of artists, finding that the trends hold even when controlled for time of joining, evidence of universality in the scaling features. We present the distributions for each group of artists in SI Fig 5 and the estimates in Table 1.

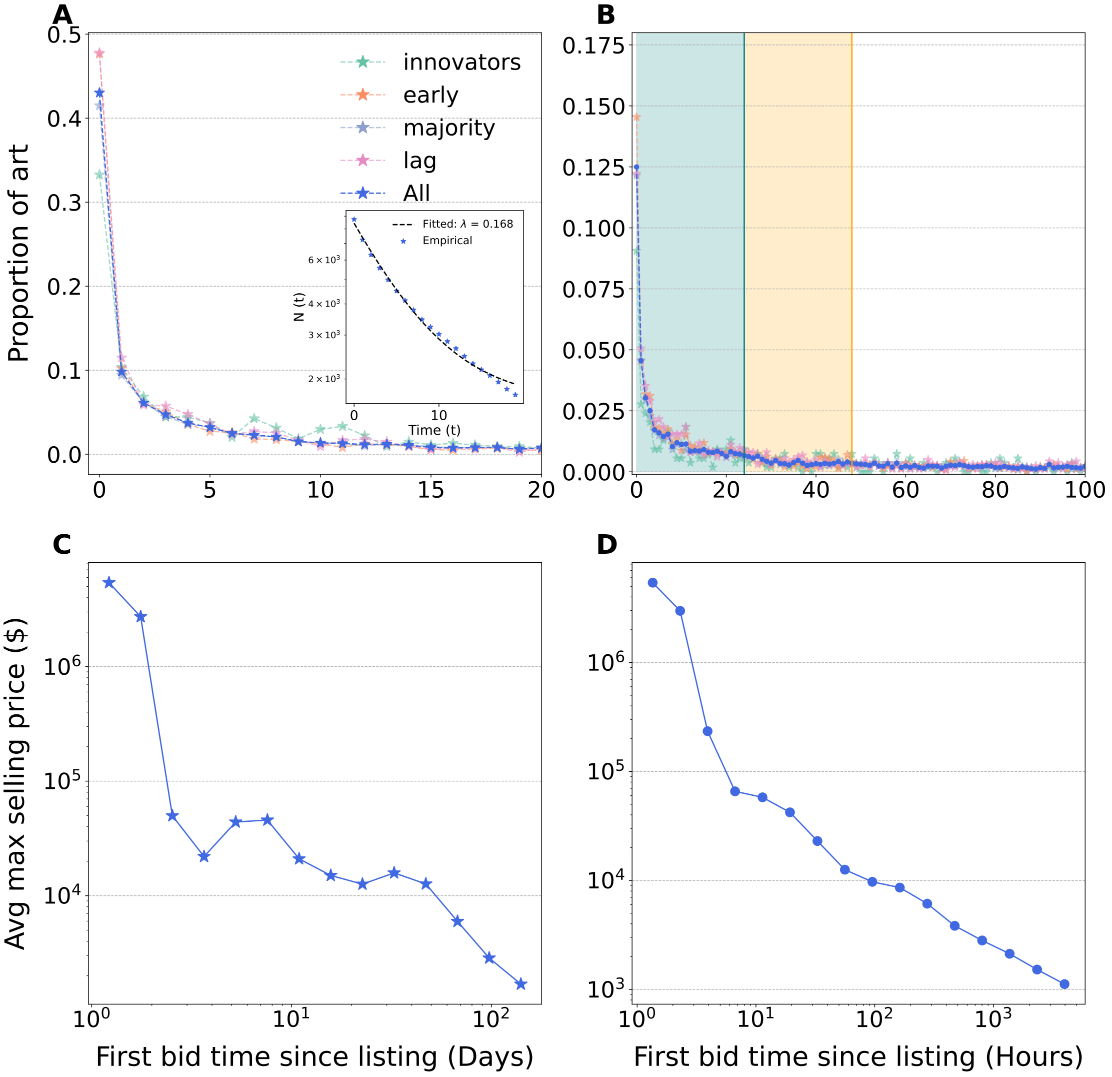

Figure 4. Activity dynamics of art. (A) Number of days taken for art to receive its first bid from the time of listing. This appears follows an exponential decay with $\lambda = 0.18$ (inset), indicating that novelty of art decays at a fast pace. (B) Time to sell (hours) from the time of listing, finding that majority of the art receives its first bid within the first few hours of listing. (C) Selling price of art based on the number of days it stays on the market before first bid, indicating that NFTs that receive early bids tend to attract high prices. (D) The price differences as a function of time to first bid since listing (hour). These findings highlight that collectors rapidly lose interest in art and an art is more likely to be sold during the first few days of its listing.

Table 1. Scaling coefficient estimates. Parenthesis represents the 95% confidence interval.

$log(earning)$	Initial Adopters	Early Majority	Majority	Laggards	**Overall**
Foundation Followers	0.851 (0.789 - 0.913)	0.894 (0.86 - 0.929)	0.709 (0.678 - 0.741)	0.575 (0.456 - 0.693)	**0.788** (0.764-0.811)
Twitter Followers	0.349 (0.259 - 0.439)	0.328 (0.286 - 0.37)	0.264 (0.245 - 0.285)	0.247 (0.181 - 0.314)	**0.305** (0.288 - 0.322)

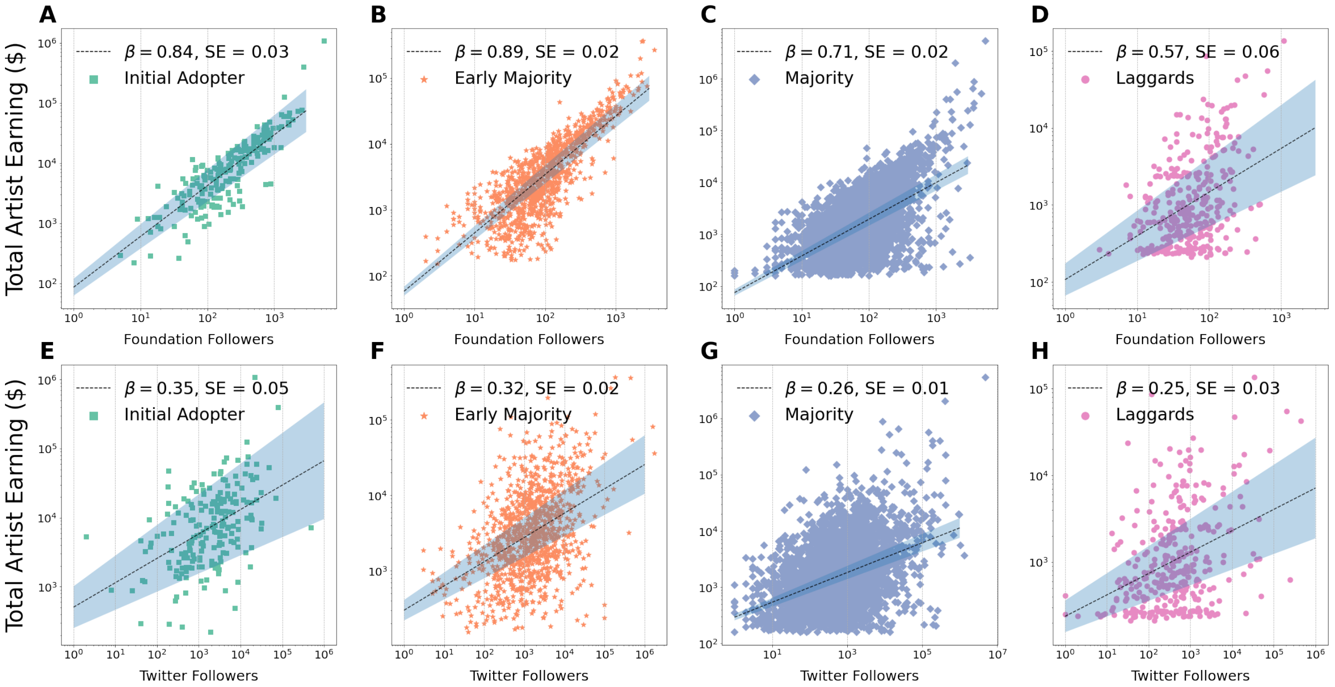

Figure 5. Followers counts versus artist earning grouped by each innovation life-cycle. (A-D) shows the total artist earning as a function of number of Foundation followers. (E-H) shows the total artist earning as a function of number of Twitter followers.

5 Artist Social Network

To list and to sell art on the platform, an artist needs to either (1) be a community upvoted artist (i.e. be popular amongst crypto art enthusiasts) representing 5% of the invites or (2) be invited by an artist or a collector, representing the remaining 95%. While (1) requires prior popularity, (2) is largely driven by offline and online social connections (Table 2). The number of clusters and the average size of clusters experienced a rapid growth during the early majority and majority period and has remained stable beginning April (SI Fig 6).

Table 2. Invites to artists across types of users. Row represents the invitee and the column represents the invited user.

User Category	Initial Adopters	Early Majority	Majority	Laggards	**Invites Sent**
Initial Adopters	137	571	504	56	1268
Early Majority	1	910	2915	246	4072
Majority	3	6	5843	1664	7516
Laggards	-	-	-	229	229
Buyers	32	196	513	93	834
**Total Invites Received**	173	1683	9775	2288	-

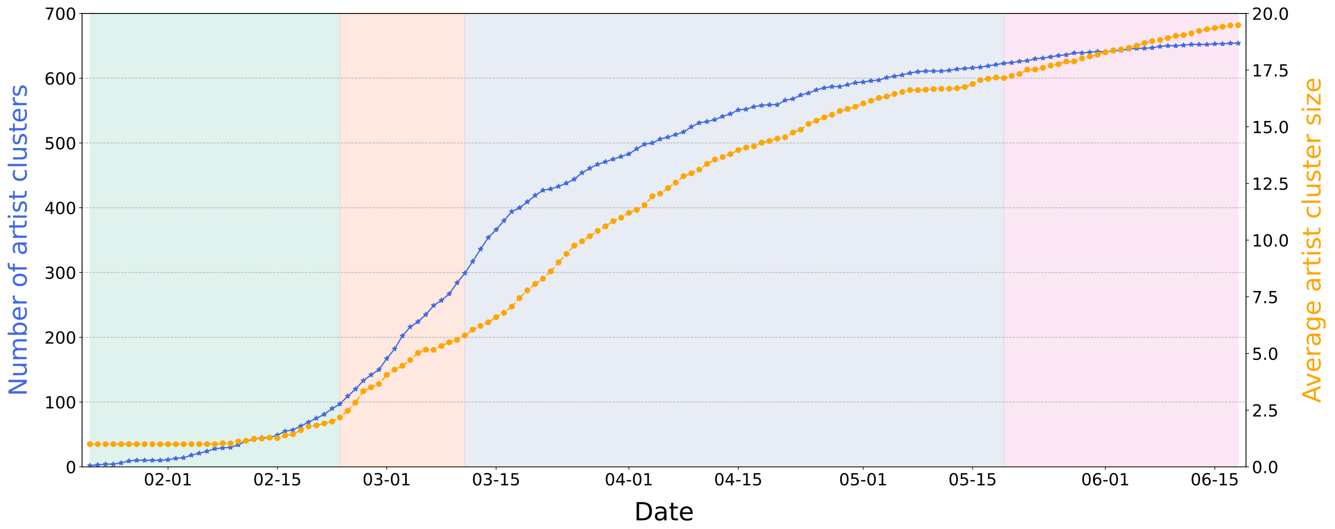

Figure 6. Growth of the artist network. We find that beginning February 15 up until end of March, the social network had a steep rise in terms of number of artist clusters and the average cluster size but has stabilized beginning June, representing the organic growth of artist clusters.

5.1 Local effect of invites

Foundation is an organically growing platform, driven by the ability of the current artists to invite new artists to the platform. The collected data offers an opportunity to ask, how similar are the invited artist to the invitor? We explore this online behavior through multiple facets of artist characteristics: total earning, number of artworks sold, number of Foundation followers, and number of Twitter followers. For each of these node attributes, we conduct random shuffling of values across all nodes and take the average of the 25 generated networks. As an additional randomized version, we also conduct link randomization while preserving the degree of the nodes, thus creating two versions of random reference. We find that artists with higher earning per art tend to invite artists with lower earning per art at a higher rate than expected at random (SI Fig 7 A). In particular, 78.76% of the invited artists had a lower earning per art than the invitor, compared to the randomized expectation of 66.59% of the artists. This translates to an average earning gap of $2,097 per art, while the random experiments estimated an average lower earning of $563.

It is important to highlight that likely the newly invited artists received lower earnings than the artist who invited them due to the temporal nature of invites. That is, the invitor joined the platform prior to the invited artist, allowing the invitor to reap benefit of strong first movers' advantage, as discussed in the manuscript. Furthermore, the difference in earnings between the invited artist and the invitor are small. These differences are even smaller in the randomized versions ($563), because by randomizing the links we remove the effect of first movers' that allowed some artists to fetch higher prices for their artworks.

Thus, our finding about the local effect of invites does not contradict the results on homophily in artist invites.

We observe similar features in the number of art sold and Foundation/ Twitter followers, where the invited artist has fewer creations and lower followers respectively than the random realizations (SI Fig 7 B-D). On average, invited artist tend to sell 2.6 (random: 1.5) fewer art, have 158 (random: 94) fewer Foundation followers, and 3,603 (random: 2,170) fewer Twitter followers. Again, these differences between artists are small and co-exist with our finding that two artists connected by invites are similar in the larger set of parameters defining artistic success. Indeed, the invited artists tend to have similar features to the artist who invited them, indicating that artists tend to invite other artists with a similar perceived earning capacity and reputation.

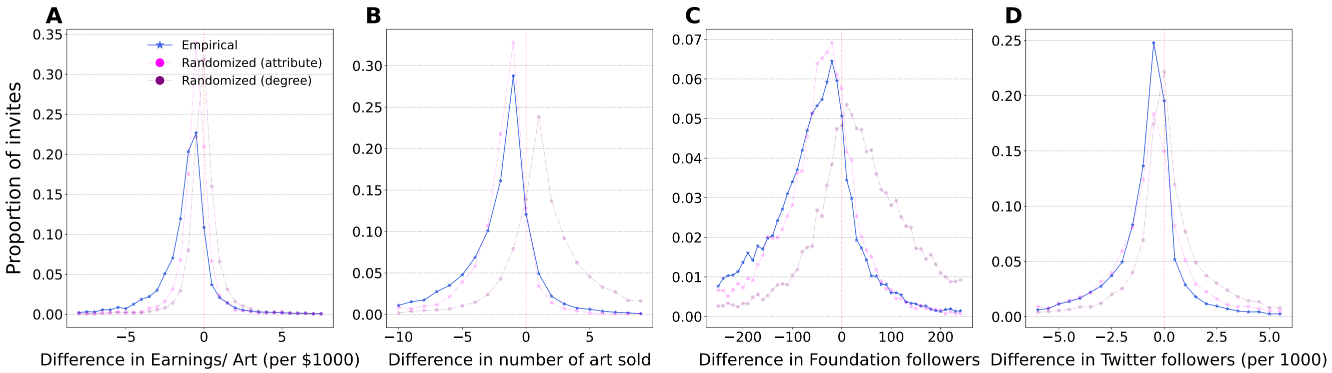

Figure 7. Characteristics of difference in invites. (A) The difference in artist earning per art based on the invitee and the invited artist, indicating homophily in artist earning. (B) Difference in number of art sold (C) Difference in number of Foundation followers (D) Difference in number of Twitter followers. We find that artists tend to invite others with a similar level of perceived reputation.

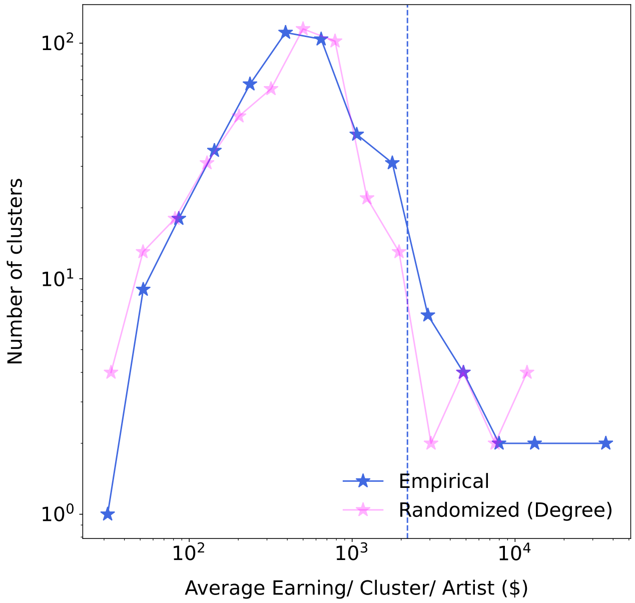

Figure 8. Controlling for one time artist sales. We remove the highest sold artwork in each cluster to evaluate the differences in average earning that is not emphasized due to one time success. The maximum earning per art per artist per cluster is $33,609 (mean: $843) and the randomized versions has a maximum earning of $13899 (mean: $677). Indeed, after removing the highest art sale in each cluster, we find rich clusters that are persistent through multiple high art sales.

6 Sustained artist reputation

6.1 Stability in earning

We explore the role of reputation and the sustained ability of high reputation artists to attract high prices. We provide examples of artists in different groups (SI Fig 9), highlighting that irrespective of the number of artworks sold, artists sell subsequent items in a range derived by the artist reputation.

6.2 Artist visibility versus maximum earning

We look at the role of artist reputation on their visibility. We find that artists with high reputation also have high followings, partially explaining their ability to repeatedly attract high prices for artworks (SI Fig 10).

6.3 Artist visibility and bidding patterns

We investigate the role of reputation on the attention received on the listed art. We find that high reputation artists not only receive high prices for the works but also attract higher number of bids (SI Fig 11). This effect remains stable throughout their career, highlighting that high reputation artists repeatedly attract higher number of bids for their works.

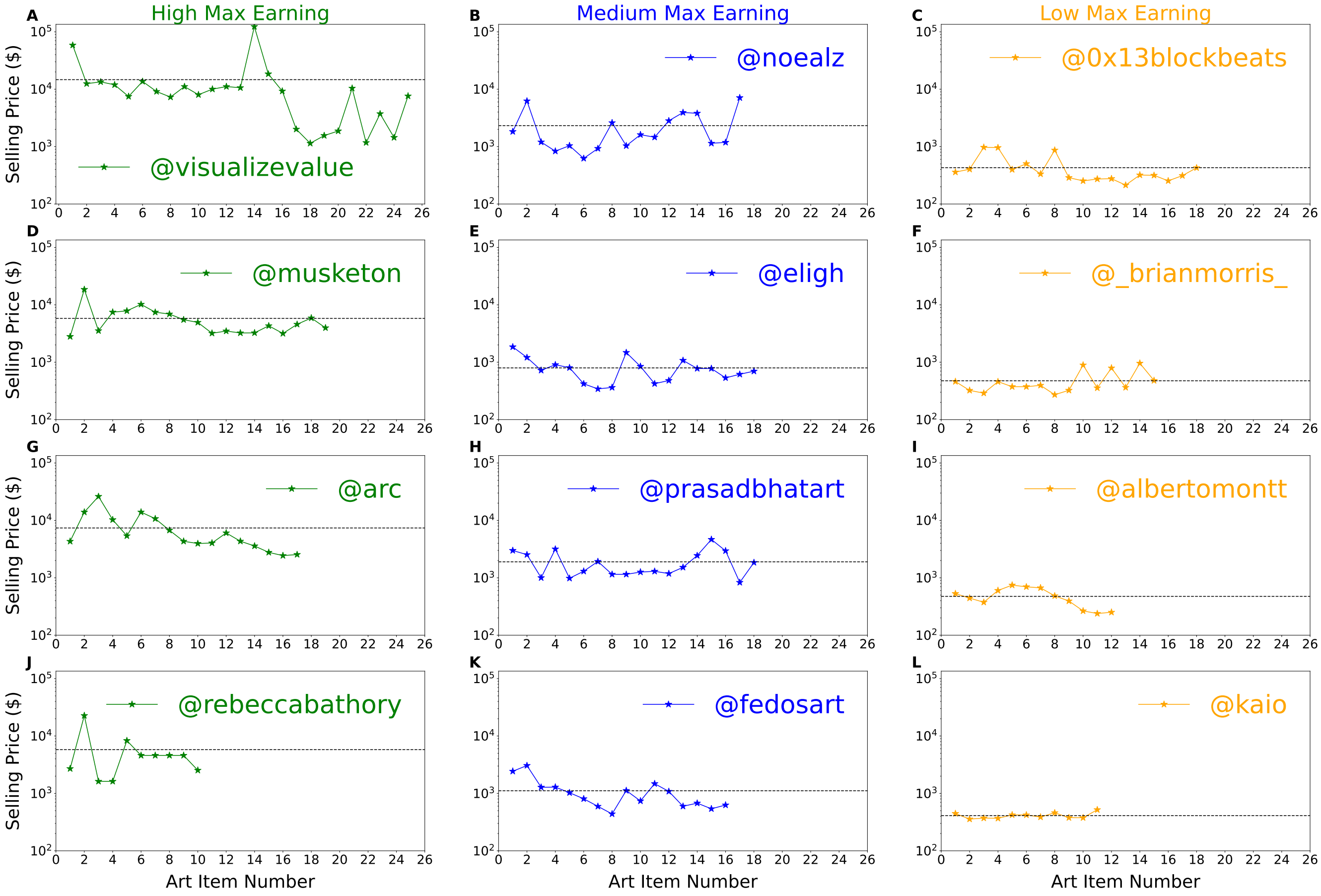

Figure 9. Stability and fluctuations in artist earning. We provide examples comparing the earning capacity for subsequent art sales for artists grouped by different reputation categories. We observe that, while prices changes significantly across subsequent art sales, they fluctuate in a comparable range that determines the artist reputation. That is high reputation artists repeatedly receive high prices for their works, while low reputation artists struggle to attract high prices.

6.4 Peak art success

The timing of highest sale (t^*, peak art price) is found to occur a random point in the career of an artist and is similar to the random career permutations (SI Fig 12 A), a pattern similar to the ones seen in scientific careers. Yet, high impact artists find their success earlier in their career compared to the medium and low impact artists (SI Fig 12 B). The average sale price of the highest sold art is significantly higher compared to the previous and subsequent art sale price (SI Fig 12 C). This indicates that there are no recognizable changes in the price leading up to and following the peak artist success.

6.5 Collector growth

The collector growth can be characterized by measuring the number of new collectors ($N_{collector}$) that invest an artist's work. In other words, it allows us to measure the effect of repeat collectors for an artist.

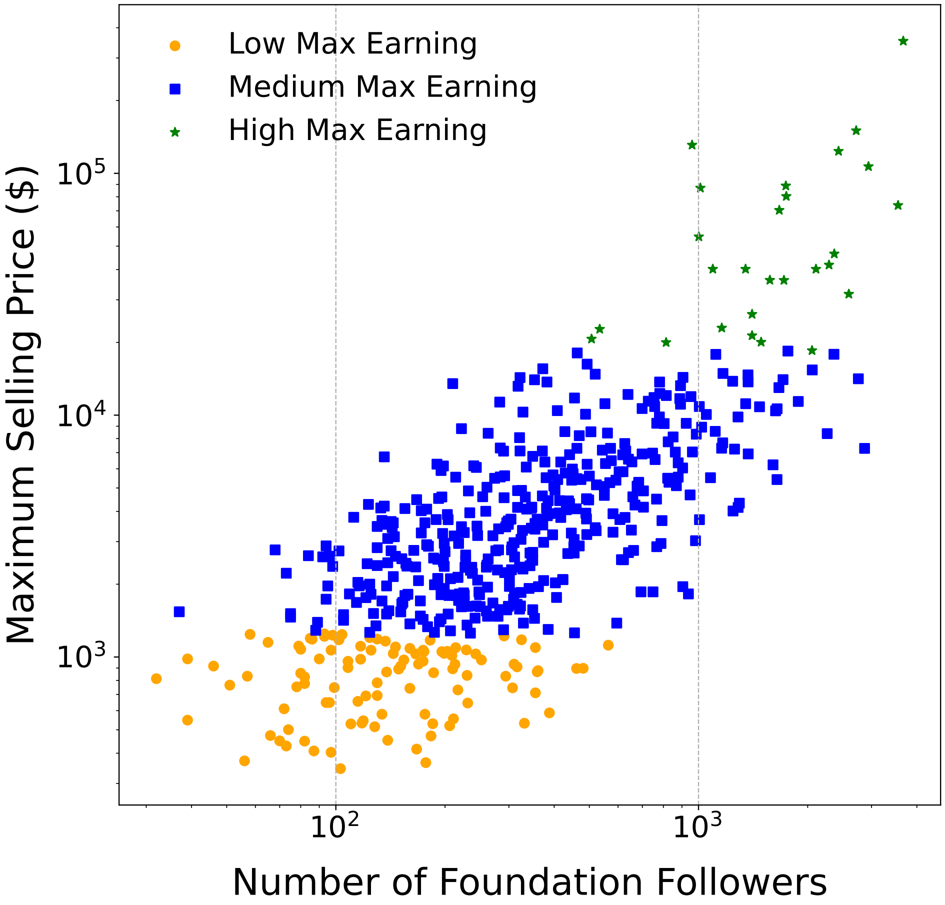

Figure 10. Artist follower counts its impact on max art sale. We find that number of Foundation followers scales linearly with the maximum selling price of art ($\beta = 0.935$), indicating that highly popular artists also attract high prices. Further, we observe a separation based on the categorized reputation level (low max earning, medium max earning, high max earning), indicating that artist reputation is correlated with artist visibility.

We find that all artists, irrespective of their reputation, acquire new buyers at a slow rate (SI Fig 13), highlighting the importance of returning collectors in artist career.

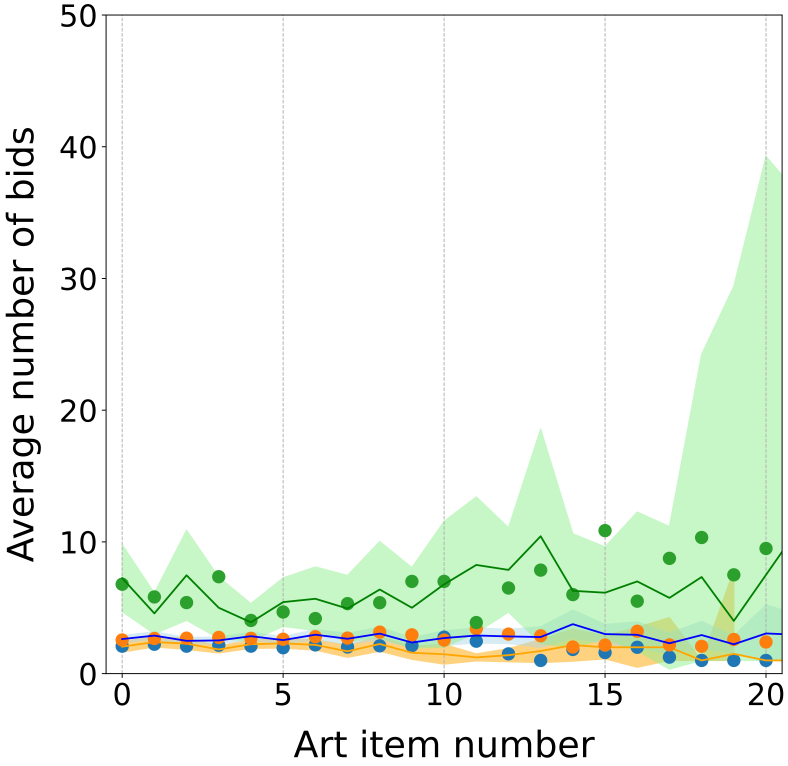

Figure 11. Artist visibility and its impact on bidding. We find that the high reputation artists continue to receive higher number of bids for their work. The shaded region represents the randomized careers (95% confidence interval) while the symbols indicate actual values.

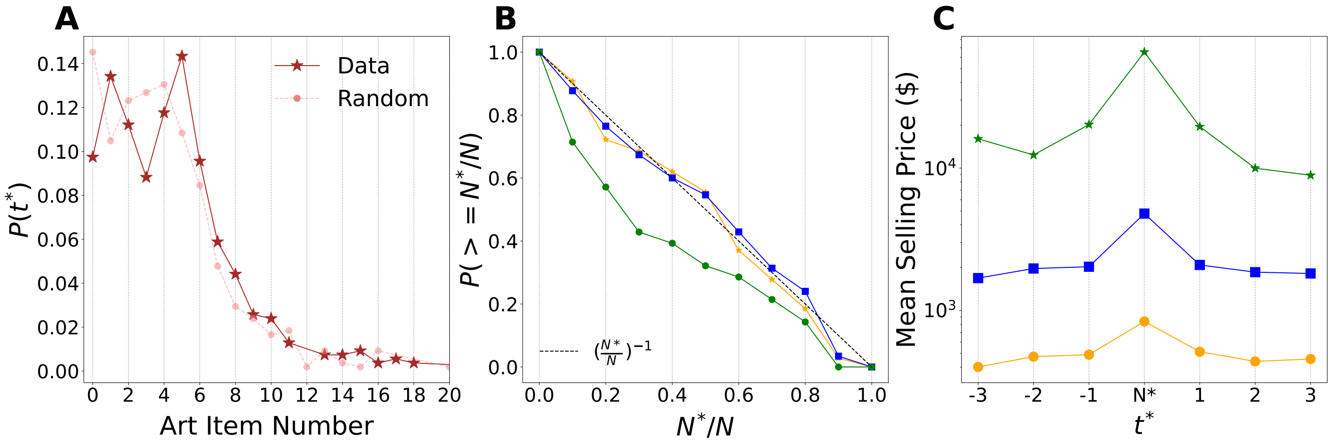

Figure 12. Timing of artist success (A) The timing of highest art sale price (t^*) for artists (star) and the random realizations (circle). We find no significant difference the timing of highest sale (Mann Whitney U Test; p val = 0.33), supporting the claim that artists may find their highest sale at any art sale. The drop off at the 6th art sale is due to the filtering of artists with atleast 6 sales. (B) Average selling price of art prior to and following the highest art sale (N^*). There exists no discernible differences in the artist success leading up to and after the highest art sale. (C) The location of the highest art sale in the artist career (N^*/N). The high impact artists appear to find their peak success early in their career compared to the medium and high impact groups.

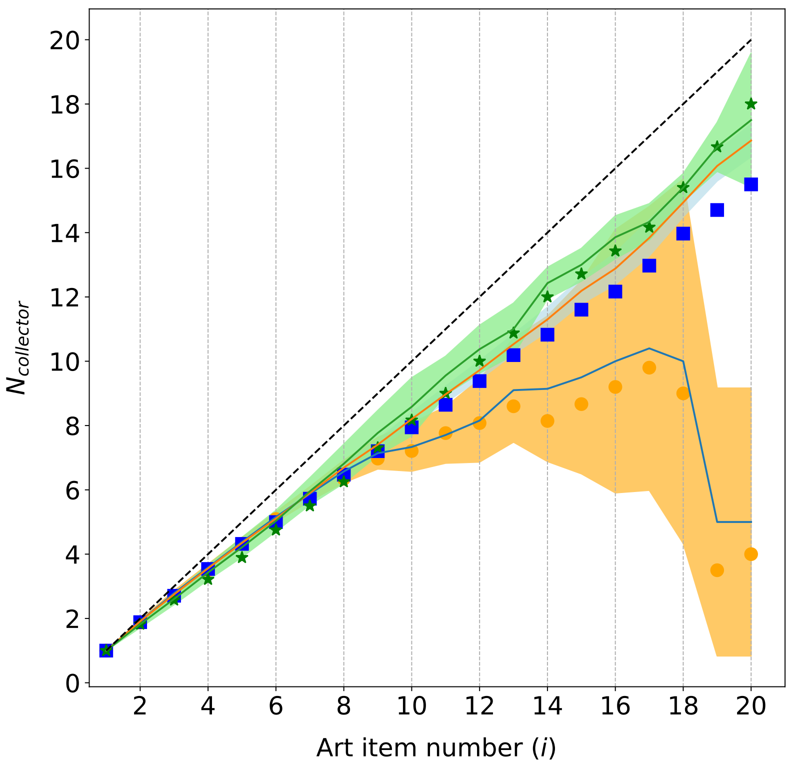

Figure 13. Artist collector growth. We find that all artists attract repeated collectors indicating a slow growth rate in the collector base. The symbols indicate the empirical average of collector growth while the shaded region show the randomized career permutations (85% confidence interval), and lines indicates the growth rate where each art is bought by a new collector.
